# The rDNA is biomolecular condensate formed by polymer–polymer phase separation and is sequestered in the nucleolus by transcription and R-loops

**DOI:** 10.1093/nar/gkab229

**Published:** 2021-04-09

**Authors:** Josh Lawrimore, Daniel Kolbin, John Stanton, Muznah Khan, Solenn C de Larminat, Colleen Lawrimore, Elaine Yeh, Kerry Bloom

**Affiliations:** Biology Department, University of North Carolina at Chapel Hill, Chapel Hill, NC 27599, USA; Biology Department, University of North Carolina at Chapel Hill, Chapel Hill, NC 27599, USA; Biology Department, University of North Carolina at Chapel Hill, Chapel Hill, NC 27599, USA; Biology Department, University of North Carolina at Chapel Hill, Chapel Hill, NC 27599, USA; Biology Department, University of North Carolina at Chapel Hill, Chapel Hill, NC 27599, USA; Biology Department, University of North Carolina at Chapel Hill, Chapel Hill, NC 27599, USA; Biology Department, University of North Carolina at Chapel Hill, Chapel Hill, NC 27599, USA; Biology Department, University of North Carolina at Chapel Hill, Chapel Hill, NC 27599, USA

## Abstract

The nucleolus is the site of ribosome biosynthesis encompassing the ribosomal DNA (rDNA) locus in a phase separated state within the nucleus. In budding yeast, we find the rDNA locus and Cdc14, a protein phosphatase that co-localizes with the rDNA, behave like a condensate formed by polymer–polymer phase separation, while ribonucleoproteins behave like a condensate formed by liquid-liquid phase separation. The compaction of the rDNA and Cdc14’s nucleolar distribution are dependent on the concentration of DNA cross-linkers. In contrast, ribonucleoprotein nucleolar distribution is independent of the concentration of DNA cross-linkers and resembles droplets *in vivo* upon replacement of the endogenous rDNA locus with high-copy plasmids. When ribosomal RNA is transcribed from the plasmids by Pol II, the rDNA–binding proteins and ribonucleoprotein signals are weakly correlated, but upon repression of transcription, ribonucleoproteins form a single, stable droplet that excludes rDNA-binding proteins from its center. Degradation of RNA–DNA hybrid structures, known as R-loops, by overexpression of RNase H1 results in the physical exclusion of the rDNA locus from the nucleolar center. Thus, the rDNA locus is a polymer–polymer phase separated condensate that relies on transcription and physical contact with RNA transcripts to remain encapsulated within the nucleolus.

## INTRODUCTION

The nucleolus is a distinct, membrane-less compartment within the nucleus that partitions both ribosomal DNA (rDNA) and the biosynthetic machinery for ribosome assembly from the rest of the nucleus. The liquid-like nature of nucleoli was first observed in *Xenopus laevis* oocytes (1), and later the nucleoli of both *X. laevis* (2) and *Caenorhabditis elegans* (3) were described as liquid–liquid phase separations. The nucleoli of human cell lines, HEK273T and HeLa, were recently found to be biomolecular condensates formed by liquid-liquid phase separation (LLPS) (4), which act as protein quality control compartments (5).

The rDNA in budding yeast is organized as a single locus on chromosome XII (6). When visualized *in vivo* using fluorescently tagged proteins that bind to the rDNA such as Net1 or Cdc14, the rDNA is encapsulated within the nucleolus (7,8). Computational simulations have shown that cross-linking within the rDNA via proteins such as condensin, is sufficient to segregate the rDNA locus from the remainder of the genome, creating a biomolecular condensate formed by polymer–polymer phase separation (PPPS) (9,10). In this context, a cross-linker is a protein or protein complex that non-covalently binds to multiple sites on the rDNA locus forming one or many chromatin loops. Recently a set of criteria that distinguish condensates formed by LLPS from condensates formed by PPPS in chromatin environments was put forth (11). Previous studies (12,13) have systematically determined the localization pattern of rDNA-binding and ribonucleolar proteins differ. In this study, we query if fluorescently labeled rDNA and ribonucleolar proteins exhibit properties of PPPS or LLPS condensates.

A condensate formed by PPPS is compressible, meaning a polymer of a fixed mass can occupy a larger or smaller volume via random reptation (thermal motion of polymer, analogous to snakes slithering about each other) or crosslinking of the polymer (11). Introducing additional chromatin crosslinks will decrease the volume of the PPPS, resulting in an increased concentration of both crosslinks and chromatin. Alternatively, a condensate formed by LLPS, is not compressible, meaning that adding more components increases the size of the condensate without altering the concentration of the components within the condensate (11). Recent studies have shown that condensates formed by LLPS where heterotypic interaction dominate can exhibit different component density based on the total component concentration in surrounding environment (14). Thus, while the concentration of a condensate formed by LLPS can vary, the component concentration of a condensate formed by LLPS should not exhibit an inverse correlation with the condensate volume. We use mean fluorescent signal intensity per voxel as a proxy for *in vivo* protein concentration inside the nucleolus. Measuring the volume and mean fluorescent signal intensity of the distributions of fluorescently labeled nucleolar proteins allows us to determine if specific components of the nucleolus are compressible, and whether compression alters nucleolar structure.

A condensate formed by PPPS will dissolve if the continuity of the DNA comprising the condensate is interrupted, while a condensate formed by LLPS will remain a stable droplet (11). The endogenous copies of chromosomal rDNA have been deleted from the budding yeast genome and replaced with high-copy, autonomous plasmids, each containing a single rDNA repeat driven by a Polymerase II promoter (GAL7) (15). The introduction of high-copy, extrachromosomal rDNA plasmids transforms the rDNA locus into its constituent monomeric units (the monomeric unit in the nucleolus is a single 9.1-kb rDNA repeat unit) eliminating the formation of a condensate via PPPS. While nucleolar organization and cell growth differs if the rDNA loci on plasmids are transcribed by Pol I versus Pol II (27), the GAL7 promotor allows for direct observation and comparison of nucleolar organization with rDNA transcription active and repressed.

The physical basis for miscibility of rDNA with the liquid-like protein components is not known. Previous work studied the influence of Pol I transcription on nucleolar organization using HeLa S3 and HCT116 cell lines. Repression of Pol I transcription via Pol I degradation or expression of a mutant Pol I both resulted in the reorganization of the nucleolus. Specifically, Pol I transcription factor RRN3, and rDNA-binding protein, UBF, localized to nucleolar caps at the periphery of the nucleolus upon mutant Pol I expression and Pol I degradation, respectively (27). In budding yeast, the rDNA locus separates from the liquid protein phase of the nucleolus upon inactivation of TORC1 induced by nutrient starvation (16). Moreover, demixing of the rDNA locus from nucleolar proteins is dependent upon condensin-mediated compaction of the rDNA locus (17). A range of TORC1 regulated processes impinge on nucleolar/rDNA structure. Using autonomous rDNA plasmids as a means to eliminate the polymeric aspects within the nucleolus we provide evidence that active transcription from these plasmids is sufficient to drive their localization to the liquid protein phase of the nucleolus.

## MATERIALS AND METHODS

### Budding yeast strains

All yeast strains used in this study are listed in Table 1. Yeast DCB190.1, JLY1048, JLY1091, DKY1000.1, DKY1001.1, DKY1003.1, DKY1004.2, DKY1014.1, KBY6317, KBY6325 and KBY9472.1 were grown in YPD (1% Yeast extract, 2% Bacto-peptone, 2% Dextrose). Yeast strains MH3342, KBY6310, KBY6315, JLY1089, JLY1090, KBY6445, KBY6446, KBY6327, KBY6329, KBY6331 and KBY6332 were grown in YCAT-GAL (YNB (Difco) 6.7 g/l, casamino acids (Difco) 20 g/l supplemented with adenine and tryptophan and 2% galactose). Yeast strains NOY988, KBY6413 and KBY6442 were grown in YPG (1% yeast extract, 2% bacto-peptone, 2% galactose).

**Table 1.** Budding yeast strains and plasmids

Strain Name	Genotype	Source
YEF 473B	Mat α trp1Δ63 leu2Δ1 ura3–52 his3Δ200 lys2–8D1	Bi and Pringle (44)
DCB190.1	Mat a trp1Δ63 leu2Δ1 ura3–52 his3Δ200 lys2–8D1, Cdc14-GFP(S65T)::kanMX6	*Harrison et al*. (45)
JLY1048	Mat a trp1Δ63 leu2Δ1 ura3–52 his3Δ200 lys2–8D1, Spc29-RFP::hphMX6, Cdc14-GFP(S65T)::kanMX6	*This study*
DKY1004.2	Mat a trp1Δ63 leu2Δ1 ura3–52 his3Δ200 lys2–8D1, Net1-GFP(S65T)::kanMX6	*This study*
KBY9472.1	Mat a trp1Δ63 leu2Δ1 ura3–52 his3Δ200 lys2–8D1, Spc29-RFP::hphMX6, Cbf5-GFP(S65T)::kanMX6	*Snider et al*. (46)
JLY1091	Mat a trp1 Δ 63 leu2 Δ1 ura3–52 his3Δ200 lys2–8D1, Rpa190-GFP(S65T)::kanMX6	*This study*
DKY1001.1	Mat a trp1Δ63 leu2Δ1 ura3–52 his3Δ200 lys2–8D1, Nop56-GFP(S65T)::kanMX6	*This study*
DKY1000.1	Mat a trp1Δ63 leu2Δ1 ura3–52 his3Δ200 lys2–8D1, Gar1-GFP(S65T)::kanMX6	*This study*
DKY1003.1	Mat a trp1Δ63 leu2Δ1 ura3–52 his3Δ200 lys2–8D1, Nop1-GFP(S65T)::kanMX6	*This study*
DKY1014.1	Mat α trp1Δ63 leu2Δ1 ura3–52 his3Δ200 lys2–8D1, Cdc14-mCherry::natMX6, Nop56-GFP(S65T)::kanMX6	*This study*
MH3342	Mat **a** ura3–52 leu2Δ1 his3Δ-200 trp1–63 lys2–301 ade2–101, rDNA-5×LacO, ura3–52::pGalL-GFPLacI::URA3, Spc29-RFP::hphMX6	*Harrison et al*. (45)
KBY6310	Mat **a** ura3–52 leu2Δ1 his3Δ-200 trp1–63 lys2–301 ade2–101, rDNA-5×LacO, ura3–52::pGalL-GFPLacI::URA3, Spc29-RFP::hphMX6, Cdc14-CFP::HIS3	*This study*
KBY6315	Mat **a** ura3–52 leu2Δ1 his3Δ-200 trp1–63 lys2–301 ade2–101, rDNA-5×LacO, ura3–52::pGalL-GFPLacI::URA3, Spc29-RFP::hphMX6, Cbf5-mCherry::kanMX6	*This study*
JLY1089	Mat **a** ura3–52 leu2Δ1 his3Δ-200 trp1–63 lys2–301 ade2–101, rDNA-5×LacO, ura3–52::pGalL-GFPLacI::URA3, Spc29-RFP::hphMX6, Net1-mCherry::kanMX6	*This study*
JLY1090	Mat **a** ura3–52 leu2Δ1 his3Δ-200 trp1–63 lys2–301 ade2–101, rDNA-5×LacO, ura3–52::pGalL-GFPLacI::URA3, Spc29-RFP::hphMX6, Nop56-mCherry::kanMX6	*This study*
KBY6445	Mat **a** ura3–52 leu2Δ1 his3Δ-200 trp1–63 lys2–301 ade2–101, rDNA-5×LacO, ura3–52::pGalL-GFPLacI::URA3, Spc29-RFP::hphMX6, Nop56-mCherry::kanMX6, LacI-GFP::HIS3(tetramer)	*This study*
KBY6446	Mat **a** ura3–52 leu2Δ1 his3Δ-200 trp1–63 lys2–301 ade2–101, rDNA-5×LacO, ura3–52::pGalL-GFPLacI::URA3, Spc29-RFP::hphMX6, Net1-mCherry::kanMX6, LacI-GFP::HIS3(tetramer)	*This study*
DCY1021	Mat α his5 leu2–3,112 ura3–50 CAN1 asp5 gal2 (form I1 rDNA::leu2 URA3+), Cdc14- GFP(S65T)::kanMX6, Spc29-RFP::hphMX6	*Hult et al*. (9)
DCY1036	Mat α his5 leu2–3,112 ura3–50 CAN1 asp5 gal2 (form I1 rDNA::leu2 URA3+), Spc29-RFP::hphMX6, Cbf5- GFP(S65T)::kanMX6	*This study*
KBY6325	Mat α ura3–1 leu2–3,112 his3–11 trp1–1 can1–100 ade2–1, LacI-GFP::HIS3(tetramer)	*This study*
KBY6327	MH3342 mated with KBY6325	*This study*
KBY6329	MH3342 mated with YEF 473B	*This study*
KBY6331	MH3342 mated with KBY6325, CBF5-mCherry::kanMX6	*This study*
KBY6332	MH3342 mated with YEF 473B, CBF5-mCherry::kanMX6	*This study*
KBY6317	Mat **a** trp1Δ63 leu2Δ1 ura3–52 his3Δ200 lys2–801, Cbf5-mCherry::natMX, Cdc14- GFP(S65T)::kanMX6	*This study*
NOY988	Mat α ade2–1 ura3–1 trp1–1 leu2–3,112 his3–11,15 can1–100 rdnΔΔ::hisG, pNOY130, single rDNA* at mid-V-R	*Oakes et al*. (15)
KBY6413	Mat α ade2–1 ura3–1 trp1–1 leu2–3,112 his3–11,15 can1–100 rdnΔΔ::hisG, pNOY130, single rDNA* at mid-V-R, Cdc14-GFP(S65T)::kanMX6 Cbf5-mCherry::natMX6	*This study*
KBY6442	Mat α ade2–1 ura3–1 trp1–1 leu2–3,112 his3–11,15 can1–100 rdnΔΔ::hisG, pNOY130, single rDNA* at mid-V-R, Nop56-GFP(S65T):: kanMX6, Net1-mCherry::natMX6	*This study*
KBY6409.1	Mat a ade2–1, ura3–1, leu2–3,112, his3–11, trp1–1, can1–100, rdnDD::hisG carrying pNOY (high copy number plasmid carrying GAL7–35S rDNA, 5S rDNA, URA3, 2m, amp) Cdc14- GFP(S65T)::kanMX6	*This study*
DKY1007.1	Mat a ade2–1, ura3–1, leu2–3,112, his3–11, trp1–1, can1–100, rdnDD::hisG carrying pNOY (high copy number plasmid carrying GAL7–35S rDNA, 5S rDNA, URA3, 2m, amp) Nop56- GFP(S65T)::kanMX6	*This study*
KBY6317 + pBL189	Mat **a** trp1Δ63 leu2Δ1 ura3–52 his3Δ200 lys2–801, Cbf5-mCherry::NatMX6, Cdc14- GFP(S65T)::kanMX6, pBL189 (2-μm plasmid, URA3)	*This study*
KBY6317 + pBB39	Mat **a** trp1Δ63 leu2Δ1 ura3–52 his3Δ200 lys2–801, Cbf5-mCherry::NatMX6, Cdc14- GFP(S65T)::kanMX6, pBB39 (2-μm plasmid, URA3, Rnh1)	*This study*
pBL189	pRS426 GPD	*Balk et al*. (34)
pBB39	pRS426 GPD, Rnh1	*Balk et al*. (34)
pFA6-GFP-KAN	pFA6a-GFP(S65T)-kanMX6	*Bahler et al*. (47)
pDH7	pFA6-CFP-HIS3	*Davis*
pKS390	pFA6a-mCherry-kanMX6	*Snaith et al*. (48)
pKS391	pFA6a-mCherry-natMX6	*Snaith et al*. (48)
pASF67	LacI-GFP::HIS3(tetramer)	*Aaron straight*

### Fluorescent imaging of budding yeast

Prior to imaging, cells with the ade2 mutation were grown to mid-log phase with excess adenine to prevent autofluorescence. KBY6413 and KBY6442 were grown in YPD and excess adenine for 3 to 5 hours prior to imaging to inhibit rDNA transcription from pNOY130 plasmids. All yeast strains were imaged on glass coverslips in liquid imaging media. Yeast strains DCB190.1, JLY1048, JLY1091, DKY1000.1, DKY1001.1, DKY1003.1, DKY1004.2, DKY1014.1, KBY6317 and KBY9472.1 were imaged in YC Complete with 2% filter sterile glucose. Strains containing only LacI-GFP (dimer) under the control of a galactose promoter, JLY1089, JLY1090, KBY6310, KBY6315, KBY6327, KBY6329, KBY6331, KBY6332, KBY6445 and KBY6446 were imaged in YC Complete with 2% filter sterile galactose. Yeast strain KBY6413 and KBY6442 were imaged in YC Complete (YNB (Difco) 6.7 g/l, casamino acids (Difco) 5 g/l supplemented with adenine, uracil and tryptophan) with 2% filter-sterile galactose and YC Complete 2% filter sterile glucose to maintain and inhibit rDNA transcription from pNOY130 plasmids, respectively. Yeast were imaged at room temperature (25°C) using an Eclipse Ti wide-field inverted microscope (Nikon) with a 100× Apo TIRF 1.49 NA objective (Nikon) and Clara charge-coupled device camera (Andor) using Nikon NIS Elements imaging software (Nikon). Fluorescent image deconvolution was performed using Huygens Essential (Scientific Volume Imaging, Hilversum, The Netherlands). All population imaging was performed by switching the channel prior to taking a Z-step. The filter set EGFP/DsRed Dichroic Mirror (86007bs) (480/20×, 565/25× and 525/40 m, 620/60 m) and ECFP/EYFP/mCherry (89006)(430/24×, 500/20×, 572/35×; 470/24 m, 535/30 m, 632/60 m) from, Chroma Technology, Bellows Falls, Vermont, USA.

### Fluorescent image cropping

Prior to image analysis the signal of interest was cropped from the original image using FIJI (18). The cropped images were split into signal channels and saved as TIFF files. All cropped images were 55 × 55 pixels (3.5 × 3.5 μm).

### Fluorescent signal homogeneity analysis

Fluorescent image stack files were read into MATLAB using MATLAB’s imread function. Image stacks were converted to sum intensity projections using MATLAB’s sum function. The background noise was filtered from the maximum intensity projections using MATLAB’s multithresh function and setting the pixels below the threshold value to NAN. The remaining intensity values were scaled by subtracting all intensity values by the minimum intensity value and then dividing each intensity value by the maximum intensity value. Thus, the foreground signal intensities were scaled between zero and one. The signal homogeneity was calculated using the algorithm described by (19). The signal homogeneity for each image was determined by calculating the gray-level co-occurrence matrix of the scaled, background-subtracted images using MATLAB’s graycomatrix function. The gray-level co-occurrence matrix is calculated by first scaling the non-NaN intensity levels of the filtered image to the integers 1 through 8. The gray-level co-occurrence matrix is then generated by counting the number of times a given pixel value is horizontally adjacent to a given pixel value. The gray-level co-occurrence matrix for each image was then converted into a homogeneity score using MATLAB’s graycoprops function. The homogeneity score measures the proximity of the distribution within the gray-level co-occurrence matrix to the diagonal, i.e. how frequently pixels of the same scaled value are adjacent to each other. Pixels with NaN values were not counted in the homogeneity analysis. This analysis was performed using the custom MATLAB functions quant_homog.m.

### Signal volume vs mean intensity correlation analysis and compression analysis

Fluorescent image stack files were read into MATLAB using MATLAB’s imread function. A background intensity value was calculated per image stack using MATLAB’s multithresh function. The volume of a signal was calculated as the number of voxels above the background intensity value. The mean intensity above background was calculated by summing all the intensity values above the background, dividing by the number voxels above background, and subtracting the threshold background value of the cellular background. Given Pearson correlations sensitivity to outliers, any image stack flagged as having an outlier in volume, in-focus area, maximum intensity projection area, sum intensity projection area, integrated intensity, or mean intensity above background were removed prior to calculating the Pearson correlation coefficient with MATLAB’s corr function. These analyses were performed with the custom MATLAB programs quant_area_vols.m and batch_vol_ints.m. The compression of a signal was calculated by dividing the mean intensity above background by the signal's volume.

### Fluorescence recovery after photobleaching analysis of ATP-Depleted budding yeast cells

FRAP analysis was performed on the same microscope as described above. All strains were incubated in YC Complete media with 0.02% sodium azide and 1 μM deoxy-glucose for 20 min before imaging. Prior to photobleaching with a Sapphire488–50 CDRH laser (Coherent), a single GFP image was taken. A 50-frame timelapse lasting for 30 s was acquired immediately following photobleaching. The recovery curve and percent recovery were calculated using the custom MATLAB programs frap_analysis_dir.m and batch_frap.m. Briefly, the pre-bleach image and the laser image were converted into binary masks using MATLAB’s multithresh function. The binary masks were used to create a bleached region to monitor for recovery and mean intensity value for the recovery region prior to photobleaching. A linear photobleaching rate was calculated using the change in intensity over time of the timelapse. A linear photobleaching rate resulting in a negative percent recovery was automatically increased until percent recovery was above zero. The photobleaching-corrected mean intensity values within the recovery region were divided by the mean intensity value for the recovery region prior to photobleaching to create a normalized recovery curve. The percent recovery was calculated as the difference between the normalized intensity value immediately prior to photobleaching and the normalized intensity value of the final frame of the timelapse.

### Rapamycin treatment

Logarithmic phase yeast were treated with Rapamycin (SIGMA) at a final concentration of 200nM from a DMSO stock for 1–2 h prior to imaging. Control cells were treated with equal volume DMSO for the same time period.

### Fluorescent signal analysis of cells lacking endogenous rDNA locus

Cropped images of cells lacking endogenous rDNA loci were read into MATLAB using imread function. The image stacks were background subtracted using MATLAB’s multithresh function to set an intensity threshold. Intensity values below the threshold were set to NANs (not a number), to remove them from subsequent analyses. The foreground signal volume was calculated by counting the number of voxels not set to NAN. The foreground signal standard deviation was calculated using MATLAB’s nanstd function. The correlation between Cdc14-GFP and Cbf5-mCherry intensities and Net1-mCherry and Nop56-GFP intensities were calculated using MATLAB’s corr function. These analyses were performed using the custom MATLAB programs noy_quant.m, noy_summary.m, and noy_summary_net1_nop56.m.

### Fluorescent signal analysis of RNase H1 overexpression mutant

Image processing and correlation analysis was performed in MATLAB as described above. Relative positional variance was calculated by determining the center of the Cbf5-mCherry signal and the distances of each voxel composing the Cdc14-GFP signal to the Cbf5-mCherry was measured. The relative positional variance is the variance of those Cdc14-to-Cbf5-center distances divided by the mean of distance between the Cdc14 signal and the center of the Cbf5 signal. Signal correlation and relative positional variance analysis was performed using the custom MATLAB programs rnase_quant.m and rnase_summary.m.

## RESULTS

### Ribonucleoproteins Cbf5 and Nop56 exhibit signal morphologies distinct from rDNA locus

**Figure 1.**
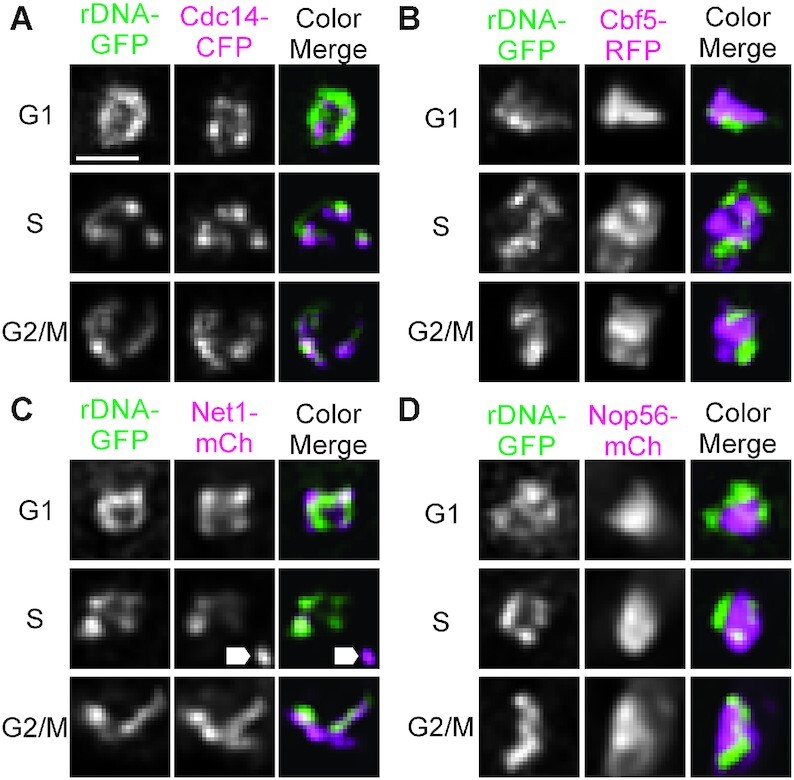
The rDNA locus co-localizes with rDNA-binding proteins Cdc14 and Net1 but not ribonucleoproteins Cbf5 and Nop56. Representative sum intensity projections of rDNA-lacO/LacI-GFP (rDNA-GFP, green) in budding yeast cells in G1, S phase and G2/M dual labeled with Cdc14-CFP (**A**), Cbf5-RFP (**B**), Net1-mCherry (**C**) and Nop56-mCherry (**D**) (magenta). Arrow heads in C indicate spindle pole body foci labeled with Spc29-RFP. Scale bar is 1 μm. Raw, whole-cell images are in Supplementary Figure S1.

The Cdc14 protein phosphatase is sequestered in the nucleolus by Net1 until anaphase onset is signaled through the FEAR (Cdc14 early anaphase release) and MEN (mitotic exit network) network (20). We fluorescently labeled Cdc14, the RENT-complex protein Net1, the pseudo-uridylate synthetase Cbf5, and the C+D snoRNP protein Nop56 in cells containing a fluorescently labeled rDNA locus (rDNA lacO/LacI-GFP) and examined their distribution in live budding yeast cells (Figure 1). As expected, the rDNA-binding proteins Cdc14 (21,22) and Net1 (8,23) exhibited morphologically similar distributions to the fluorescently labeled lacO/LacI-GFP rDNA locus during G1, S and G2/M (Figure A and C). In contrast, Cbf5 and Nop56 exhibited signals that appeared morphologically distinct from the rDNA locus signal.

### Cdc14 signals are more heterogeneous and exhibit greater compaction than Net1 and ribonucleoprotein signals

**Figure 2.**
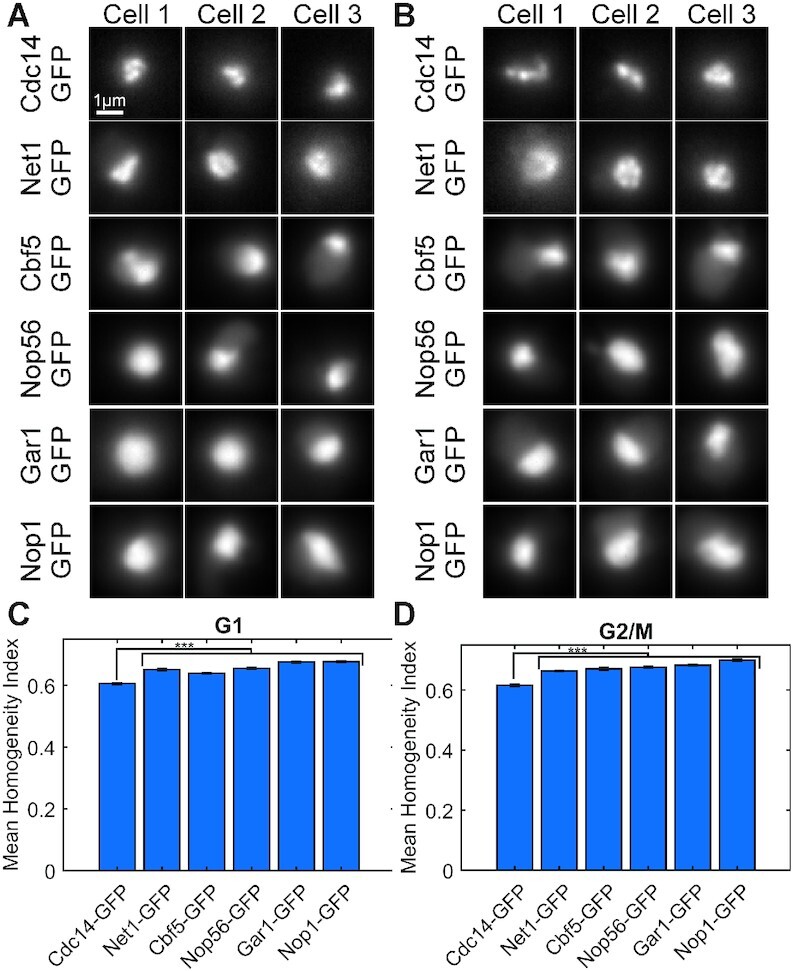
The rDNA-binding protein Cdc14 displays less signal homogeneity than Net1 and ribonucleoproteins. Representative sum intensity projections of budding yeast cells in G1 (**A**) and G2/M (**B**). Bar charts comparing the mean signal homogeneity index of budding yeast cells in G1 (**C**) and G2/M (**D**). Error bars are SEM. G1 cells, Cdc14-GFP *n* = 193, Net1-GFP *n* = 1001, Cbf5-GFP *n* = 115, Nop56 *n* = 343, Gar1-GFP *n* = 304, Nop1-GFP *n* = 338. G2/M cells, Cdc14-GFP *n* = 132, Net1-GFP *n* = 698, Cbf5-GFP *n* = 78, Nop56 *n* = 252, Gar1-GFP *n* = 307, Nop1-GFP *n* = 184. The *** indicates *P*-value <0.001 by Tukey test.

Fluorescently labeled Cdc14-GFP and Net1-mCh are markedly heterogeneous, exhibiting a punctate structure with variation in size and morphology of the distinct puncta (Figures 1A and 2A). In contrast, the ribonucleoproteins Nop56, Gar1, Nop1 and Cbf5 exhibit homogeneous signals but do vary in their spheroidal or oblate morphology (Figures 1A and 2A). The degree of homogeneity for these components was quantified using a homogeneity score that measures how frequently pixels of the same scaled value are proximal to one another (see methods). In this way, we found that Cdc14 was significantly less homogeneous than Net1 and the suite of ribonucleoproteins Nop56, Gar1, Nop1 and Cbf5 (Figure 2).

### Cdc14 signals are compressible in G1 and G2/M

**Figure 3.**
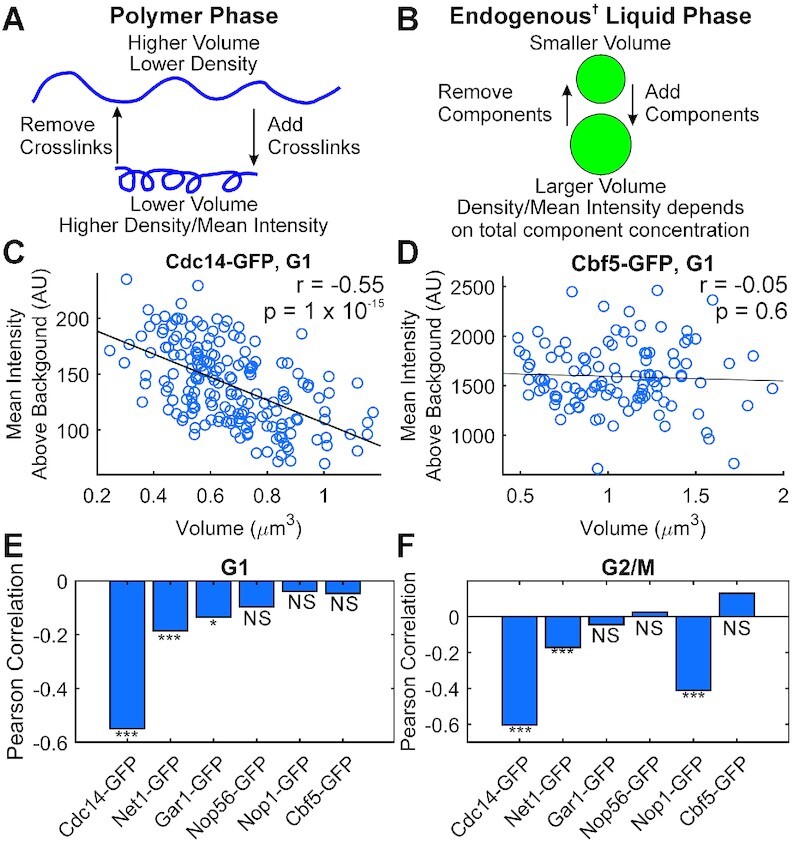
Cdc14 and Net1 signals are compressible in G1 and G2/M (**A**) Cartoon illustrating that altering number of crosslinks/loops within a polymer will alter the polymer's volume and density. (**B**) Cartoon illustrating that adding or removing components from a liquid phase will alter the liquid droplet's volume but not the droplet's density. (**C**, **D**) Scatter plots of the mean intensity above background versus volume of Cdc14-GFP signals (C) and Cbf5-GFP signals from budding yeast cells in G1 (D). Bar charts comparing Pearson's correlation coefficient of signal volume and mean signal intensity above background for budding yeast cells in G1 (**E**) and G2/M (**F**). Due to the sensitivity of Pearson's correlation to outliers, all datasets had outliers removed (see methods). G1 cells, Cdc14-GFP *n* = 185, Net1-GFP *n* = 975, Gar1-GFP *n* = 291, Nop56 *n* = 329, Nop1-GFP *n* = 328, Cbf5-GFP *n* = 112. G2/M cells, Cdc14-GFP *n* = 124 Net1-GFP *n* = 657, Gar1-GFP *n* = 297, Nop56-GFP *n* = 244, Nop1-GFP *n* = 178, Cbf5-GFP *n* = 77. The * indicates *P* < 0.05, the *** indicates *P* < 0.001, and the NS indicates *P* > 0.05 that correlation is significantly different than 0. † Endogenous Liquid Phase refers to a LLPS condensate that is dominated by heterotypic interactions. In these LLPS condensates the component concentration in the dense phase depends on the total component concentration (14).

To determine the physical characteristics of nucleolar components we analyzed the amount of protein (mean signal intensity) versus the volume occupied (Figure 3B). Representative scatter plots comparing the signal volumes and mean signal intensities of nucleolar components in G1 cells show a significant anti-correlation for Cdc14-GFP signals but no correlation for Cbf5-GFP signals (Figure 3C and D). Applying the volume vs mean intensity correlation analysis to the Net1, Gar1, Nop56 and Nop1 revealed, as observed in the homogeneity analysis, Cdc14 exhibited a much stronger anti-correlation than any of the other signals in both G1 and G2/M cells.

### Compaction of rDNA locus compacts Cdc14 and Net1 distributions but not ribonucleoproteins

**Figure 4.**
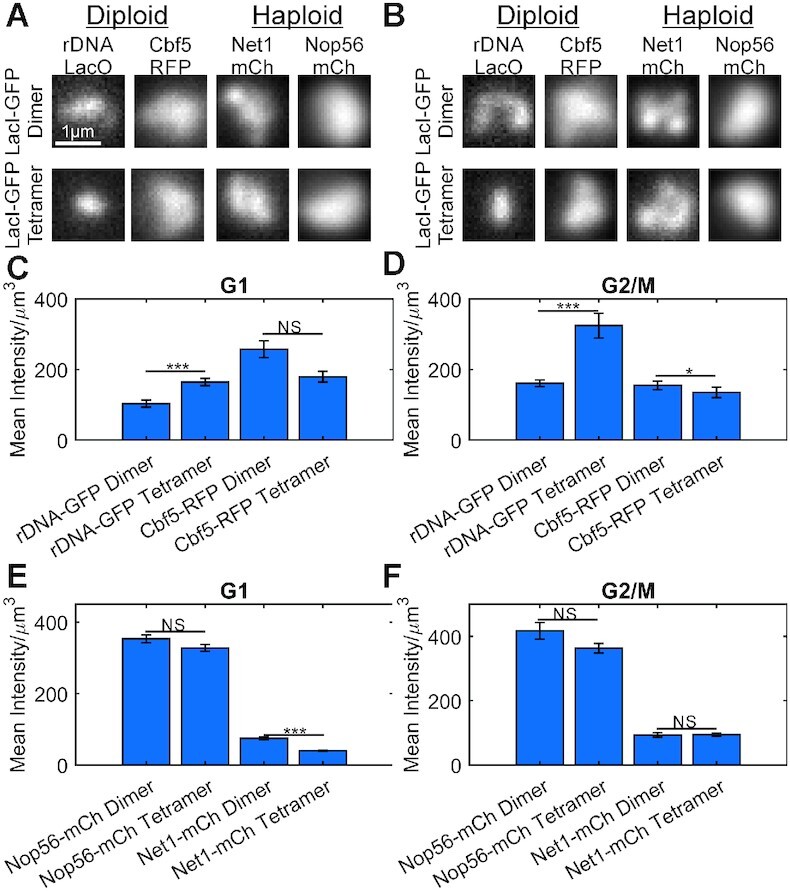
Compression of the rDNA locus does not compress Cbf5, Net1, nor Nop56 distributions. Representative sum intensity projections of budding yeast cells expressing a dimeric LacI-GFP (dimer) or a tetrameric LacI-GFP (tetramer) for budding yeast cell in G1 (**A**) or G2/M (**B**). Bar charts comparing the mean intensity above background divided by the signal volume for diploid cells in G1 (**C**) and G2/M (**D**). Bar charts comparing the mean intensity above background divided by the signal volume for haploid cells in G1 (**E**) and G2/M (**F**). Error bars are SEM. The *** indicates a *P*-value < 0.001 and NS indicates a *P*-value > 0.05 for Wilcoxon ranksum test. All strains, except for rDNA-GFP-Dimer, which was grown and imaged in media containing galactose, were grown and imaged in media containing glucose. Diploid strains, G1: rDNA-GFP Dimer n = 82, rDNA-GFP Tetramer *n* = 86, Cbf5-RFP dimer *n* = 102, Cbf5-RFP tetramer *n* = 107. Diploid strains, G2/M : rDNA-GFP dimer *n* = 91, rDNA-GFP tetramer *n* = 78, Cbf5-RFP dimer *n* = 119, Cbf5-RFP tetramer *n* = 81. Haploid strains, G1: Nop56-RFP dimer *n* = 78, Nop56-RFP tetramer *n* = 98, Net1-RFP dimer *n* = 47, Net1-RFP tetramer *n* = 156. Haploid strains, G2/M: Nop56-RFP dimer *n* = 21, Nop56-RFP tetramer *n* = 41, Net1-RFP dimer *n* = 24, Net1-RFP tetramer *n* = 54.

Given the ribonucleoprotein signals are more homogenous (Figure 2) and exhibit weaker anti-correlations than the rDNA binding protein Cdc14 (Figure 3) we wished to query if compaction of the rDNA locus would translate into compacted ribonucleoprotein signals. The *Escherichia coli* lac repressor (LacI) form dimers to bind to a single LacO site. LacI dimers can concatenate with another dimer pair bound to a different LacO site to effectively create a DNA loop if the native tetramerization domain is intact (24). We generated two diploid yeast strains and two haploid yeast strains in which LacO arrays were integrated adjacent to each 35s rDNA repeats on chromosome XII (13). The disparate LacO sequences will be crosslinked by tetrameric LacI, compressing the rDNA locus. The strains also contain a LacI-GFP fusion protein under the control of a galactose promoter that lacked the tetramerization domain (pGAL-LacI-dimer-GFP). Expression of LacI-tetramer-GFP was sufficient to compact the rDNA locus. We imaged a diploid yeast strain in galactose (Dimer expression) or glucose (Tetramer expression) to measure the compaction (mean intensity/volume) of rDNA loci fluorescently labeled with pGAL-LacI- GFP. The rDNA locus labeled with LacI-tetramer-GFP (glucose-grown) was significantly more compact than the fluorescently labeled LacI-dimer-GFP (galactose) in G1 and G2/M cells (Figure 4A-D). We imaged yeast strains containing Cbf5-RFP, Net1-mCherry and Nop56-mCherry in media containing glucose (LacI-tetramer-GFP expression), and found that unlike the rDNA locus itself, neither Net1, Cbf5, nor Nop56 signals exhibited significant compaction upon compaction of the rDNA locus (Figure 4).

**Figure 5.**
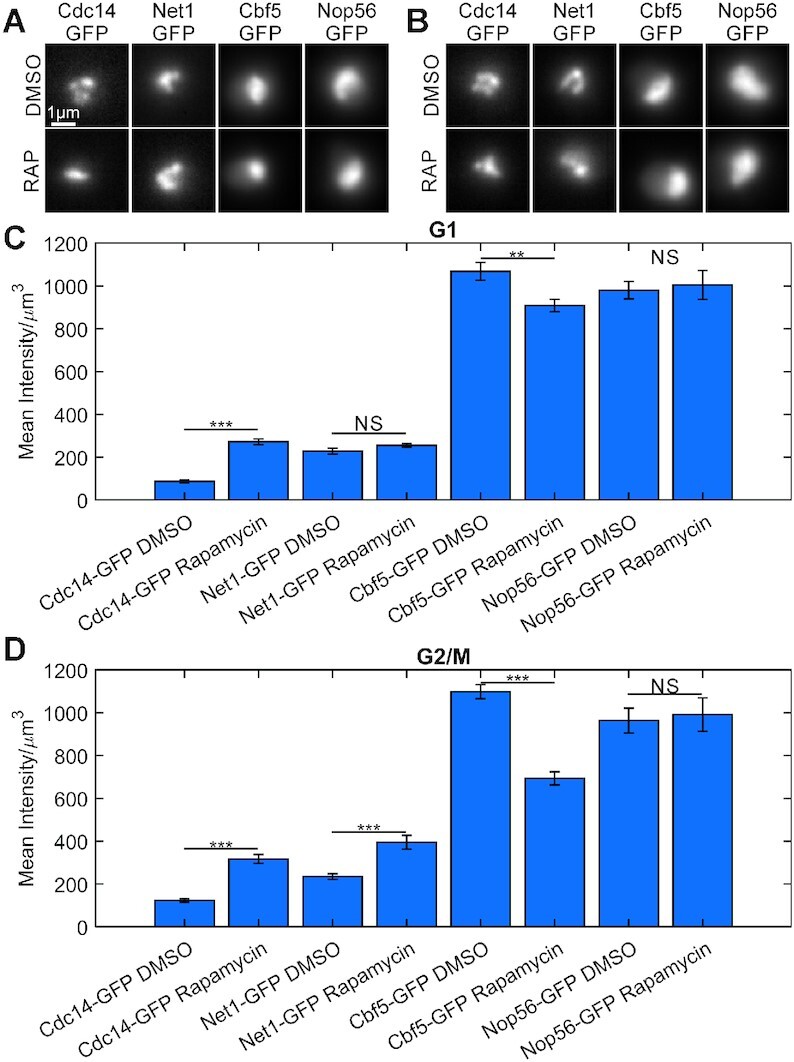
Cdc14 and Net1, but not ribonucleoprotein, signals compress upon rapamycin treatment. Representative sum intensity projections of budding yeast cells treated with DMSO or rapamycin in G1 (**A**) or G2/M (**B**). Scale bar is 1 μm. Bar charts comparing the mean intensity above background divided by signal volume in G1 (**C**) and G2/M (**D**) cells. Error bars are SEM. G1 cells, Cdc14-GFP DMSO *n* = 34, Cdc14-GFP Rapamycin *n* = 47, Net1-GFP DMSO *n* = 54, Net1-GFP Rapamycin *n* = 115, Cbf5-GFP DMSO *n* = 44, Cbf5-GFP Rapamycin *n* = 98, Nop56-GFP DMSO *n* = 21, Nop56-GFP Rapamycin *n* = 23. G2/M cells, Cdc14-GFP DMSO *n* = 46, Cdc14-GFP Rapamycin *n* = 51, Net1-GFP DMSO *n* = 85, Net1-GFP Rapamycin *n* = 60, Cbf5-GFP DMSO *n* = 60, Cbf5-GFP Rapamycin *n* = 43, Nop56-GFP DMSO *n* = 15, Nop56-GFP Rapamycin *n* = 20. The *** indicates a *P*-value <0.001, ** indicates a *P*-value <0.01, and NS indicates a *P*-value >0.05 for Wilcoxon ranksum test.

An alternative strategy to compact the rDNA is through rapamycin inhibition of the TOR kinase pathway (25). Cdc14-GFP signals showed a significant increase in compaction in G1 cells and G2/M cells deduced from increased intensity in a smaller volume, (Figure 5A–D), while Net1-GFP showed a significant increase in compaction in G2/M cells (Figure 5D). In contrast, Cbf5 and Nop56 signals did not exhibit a change in compaction in either G1 or G2/M cells (Figure 5C and D). Cdc14-GFP appears to display the compaction reported for the rDNA locus while Net1-GFP only exhibits compaction in G2/M cells.

### Net1, Polymerase subunit RPA190 and Cbf5 signals exhibit faster kinetics than rDNA locus in ATP-depleted cells

**Figure 6.**
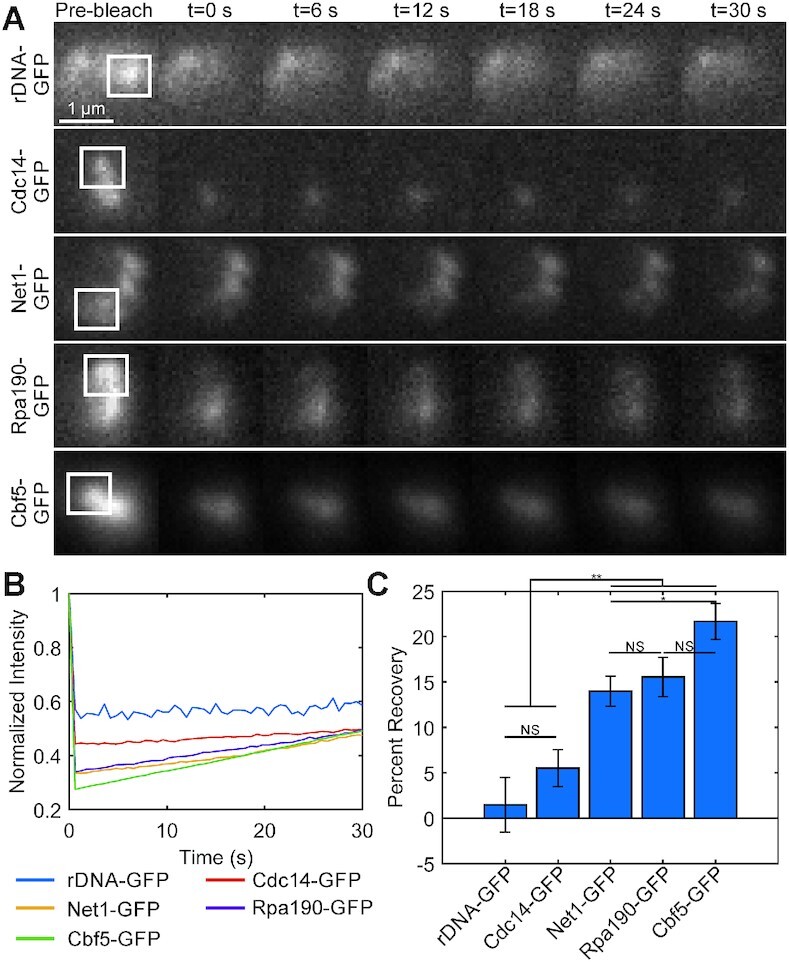
Fluorescently labeled rDNA-lacO/LacI-GFP locus and rDNA-binding protein Cdc14-GFP exhibit slower turnover than Net1-GFP, Rpa190-GFP and Cbf5-GFP. (**A**) Representative montages of FRAP experiments of ATP-depleted budding yeast cells in G1. Arrows indicate photobleached region. Scale bar is 1 μm. (**B**) Line plots comparing mean normalized recovery curves. (**C**) Bar chart comparing mean percent recovery. Error bars are SEM. rDNA-GFP *n* = 12, Cdc14-GFP *n* = 26, Net1-GFP *n* = 40, Rpa190-GFP *n* = 23, and Cbf5-GFP *n* = 28. The ** indicates *P* < 0.01 and NS indicates *P* > 0.05 by Tukey test.

Net1 and Cdc14 are considered to be a bona fide rDNA locus markers. Our findings that Net1 and Cdc14 exhibit differential signal homogeneity (Figure 2) and cell cycle compaction (Figures 4-5) reveal additional complexity. It has been found that Net1 coimmunoprecipitates with Rpa190, the largest subunit of POL 1 (26). If a subpopulation of Net1 was bound to Rpa190 and sequestered with ribonucleoproteins then Net1 may exhibit physical properties of both compressible and non-compressible compartment. To distinguish if Net1 signal behavior reflects rDNA locus behavior we performed fluorescent recovery after photobleaching (FRAP) analysis on rDNA-LacI-GFP (dimeric LacI-GFP), Cdc14-GFP, Rpa190-GFP and Cbf5-GFP in cells depleted of ATP via sodium azide and deoxyglucose treatment. ATP-depletion depresses chromatin motion and prevents turnover of LacI-GFP from the lacO binding site, so any recovery after photobleaching should reflect protein dynamics (27–29). Thus, while the FRAP experiments do not reflect the motion of an unperturbed yeast nucleolus, the motion we observe in ATP-depleted cells better represents the physical properties of the rDNA and nucleolus as the motion is not confounded with ATP-dependent processes. The rDNA binding LacI-GFP and Cdc14 signals exhibited significantly less recovery than Net1, Rpa190 and Cbf5 signals (Figure 6). The percent recovery was not significantly different between Net1 and Rpa190 signals, while Cbf5 signals exhibited the highest percent recovery (Figure 6C). Thus, Net1 signal dynamics does not reflect the turnover of rDNA binding proteins in ATP-depleted cells, consistent with two pools of Net1 in the nucleolus.

### Dissolution of the endogenous rDNA reveal transcriptional requirement for rDNA localization with ribonucleoproteins

**Figure 7.**
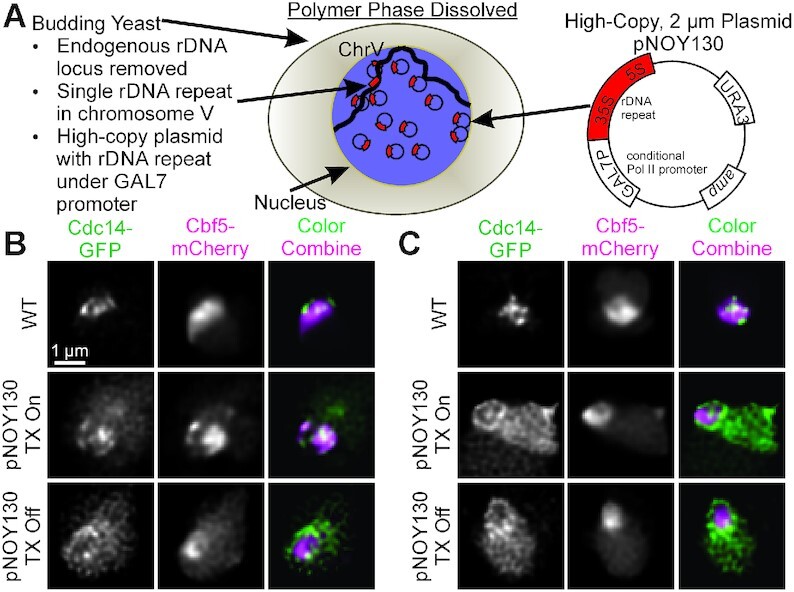
Cdc14-GFP is excluded from Cbf5-mCherry in strains with and without intact rDNA locus upon loss of transcription. (**A**) Cartoon illustrating the budding yeast strain NOY988. The endogenous rDNA locus was replaced with a high-copy plasmid containing a single rDNA repeat under a pGAL7 promoter and a single rDNA repeat in chromosome V. Deconvolved, representative images of yeast labeled with Cdc14-GFP (green) and Cbf5-mCherry (magenta) in G1 (**B**) and G2/M (**C**). Scale bar is 1 μm.

To mimic a dissolved rDNA locus, we fluorescently labeled proteins within the budding yeast strain, NOY988, containing a high-copy 2-μm plasmid containing the 35S and 5S rDNA genes under the control of a Polymerase-II-transcribed, galactose promoter (pNOY130). The endogenous rDNA locus on Chromosome XII has been deleted but the strain contains a single, ectopic rDNA repeat in middle of chromosome V-R (15,30). Given the rDNA locus is present as 40–60 extra-chromosomal monomers and a single rDNA repeat on chromosome V, we have severed the continuity of the rDNA in its host configuration as a contiguous polymer, effectively dissolving the rDNA locus (Figure 7A). Previous work has shown that nucleolar organization differs if rDNA plasmids are transcribed by polymerase I or by polymerase II (30), but the galactose promotor allows for direct repression of rDNA transcription from the plasmids. Recent studies have shown that inhibition of TORC1 in budding yeast physically separates the rDNA from nucleolar proteins during nucleophagy (16,17). Given most of rDNA transcription in NOY988 is Pol-II-dependent and repressible, we also grew NOY988-derived strains to media containing glucose to determine if preventing the rDNA locus from forming a PPPS condensate interferes with the separation of rDNA-binding proteins from nucleolar proteins.

**Figure 8.**
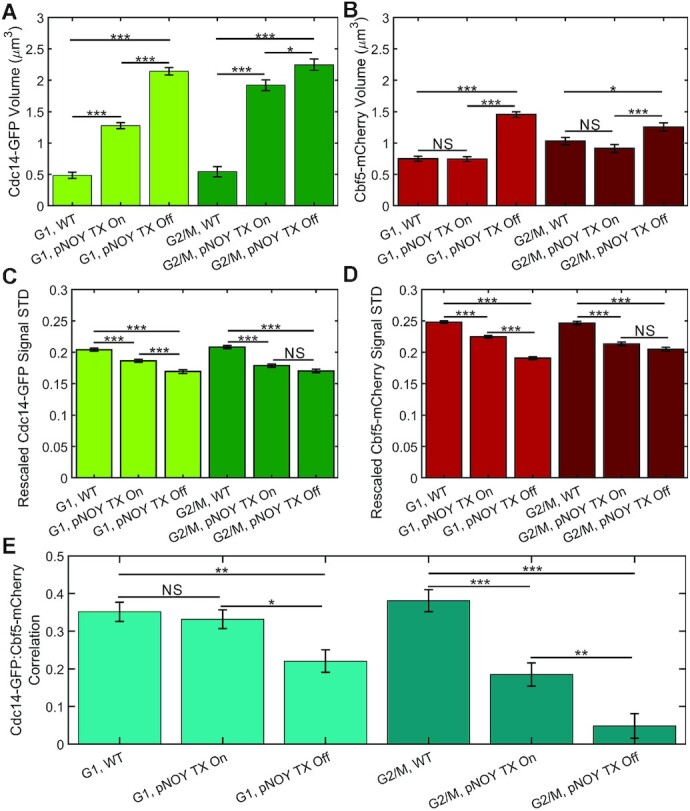
Cdc14 and Cbf5 signals expand, homogenize and separate upon loss of transcription in cells lacking a contiguous rDNA locus. Bar charts comparing the signal volume of Cdc14-GFP (**A**) and Cbf5-mCherry (**B**) of budding yeast cells in G1 and G2/M. Bar charts comparing the rescaled signal standard deviation of Cdc14-GFP (**C**) and Cbf5-mCherry (**D**). Signal intensity values were scaled between 0 and 1 prior to standard deviation calculation. (**E**) Bar chart showing mean Pearson correlation coefficient of Cdc14-GFP and Cbf5-mCherry signal intensities. G1 cells, WT *n* = 87, Tx on *n* = 93, Tx off *n* = 63. G2/M cells, WT *n* = 67, Tx on *n* = 61, Tx off *n* = 54. The *** indicates *P* < 0.001, ** indicates *P* < 0.01, * indicates *P* < 0.05, and NS indicates *P* > 0.05 by Tukey test.

**Figure 9.**
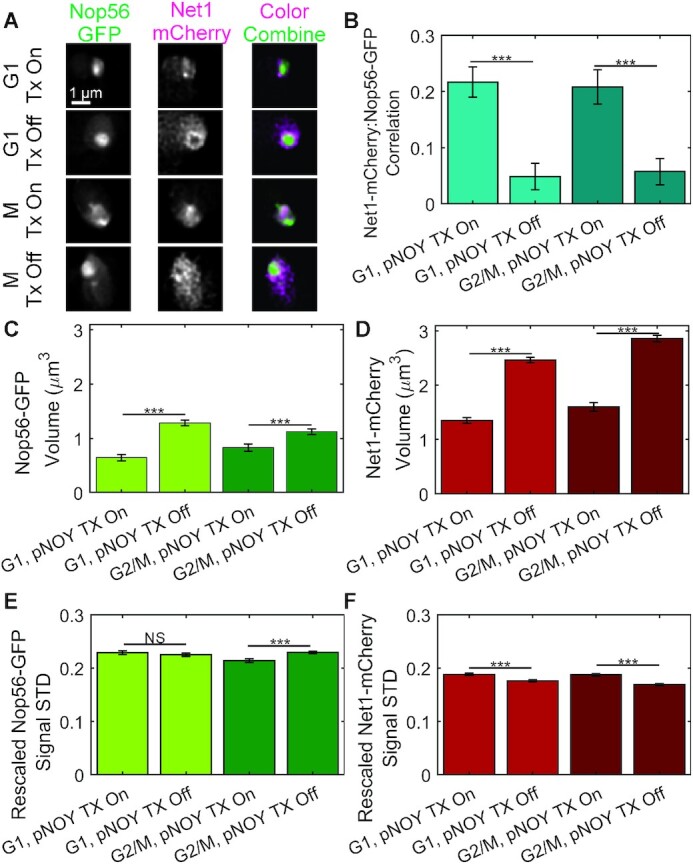
Net1-mCherry signals expand, homogenize and separate from Nop56-GFP signals upon loss of transcription in cells lacking a contiguous rDNA locus. (**A**) Representative images of Nop56-GFP and Net1-mCherry in cells where the endogenous rDNA locus was replaced with high-copy plasmid containing a single rDNA repeat under a pGAL7 promoter and a single rDNA repeat in chromosome V. Scale bar is 1 μm. (**B**) Bar chart comparing Pearson correlation values of Net1-mCherry and Nop56-GFP signals of budding yeast cells in G1 and G2/M. Bar charts comparing the volume of Nop56-GFP (**C**) and Net1-mCherry (**D**) signals. Bar charts comparing the rescaled signal standard deviation of Nop56-GFP (**E**) and Net1-mCherry (**F**) signals. Signal intensity values were scaled between 0 and 1 prior to standard deviation calculation. G1 Tx on *n* = 65, G1 Tx off *n* = 84, G2/M Tx on *n* = 60, G2/M Tx off *n* = 101. The *** indicates a *P*-value < 0.001 and NS indicates a *P*-value > 0.05 for Wilcoxon ranksum test.

We transformed NOY988 with both Cdc14-GFP and Cbf5-mCherry to determine if their distributions would become more diffuse, indicative of a PPPS condensate, or form a stable droplet, indicative of an LLPS condensate and if the signals would separate upon transcriptional repression. We imaged both Cdc14-GFP and Cbf5-mCherry in live yeast cells and deconvolved the images using Huygens Essential (Scientific Volume Imaging, Hilversum, The Netherlands) and observed a striking absence of Cdc14-GFP and Cdb5-mCherry signal colocalization upon transcriptional repression (Figure 7B). Moreover, cells contained highly mobile mini-nucleoli when transcription was active (Supplementary Figure S2) but were not visible when transcription was repressed (Supplementary Figure S3). These mini-nucleoli and their dissipation was previously observed using similar high-copy rDNA plasmid in cells containing Gar1-GFP (31). Cdc14-GFP signal volumes were larger in the pNOY130-containing strain than the WT strain in both G1 and G2/M cells (Figure 8A). However, Cbf5-mCherry signal volumes were not significantly different between the pNOY130-containing strain and the WT strain in G1 and G2/M cells (Figure 8B). Transcriptional repression of pNOY130 increased Cdc14 and Cbf5 signal volumes (Figure 8A and B). Cdc14-GFP and Cbf5-mCherry signal heterogeneity was calculated as the standard deviation of the signal after rescaling the signal intensity range between zero and one. Both Cdc14-GFP and Cbf5-mCherry signals were less heterogeneous in the pNOY-containing strain than in the WT strain (Figure 8C and D). Transcriptional repression of pNOY130 decreased the signal heterogeneities of Cdc14-GFP and Cbf5-mCherry in G1 cells but did not significantly altered the signal heterogeneities in G2/M cells (Figure 8C and D). Cdc14-GFP/Cbf5-mCherry signal correlation was not significantly different between the pNOY130-containing strain and the WT strain in G1 cells but was significantly less in is G2/M cells (Figure 8E). Transcriptional repression of pNOY130 significantly lowered Cdc14-GFP/Cbf5-mCherry signal correlation in both G1 and G2/M cells (Figure 8E). We confirmed these observations by constructing, imaging and analyzing a NOY988 strain containing Net1-mCherry and Nop56-GFP. We again observed a striking separation of rDNA-binding protein Net1-mCherry from ribonucleoprotein Nop56-GFP (Figure 9A) and a drop in Net1-mCherry/Nop56-GFP signal correlation (Figure 9B) upon transcriptional repression in both G1 and G2/M cells. Upon transcriptional repression Nop56-GFP and Net1-mCherry signal volumes greatly increased in both G1 and G2/M cells (Figure 9C and D). Nop56-GFP signal heterogeneity did not significantly change upon transcriptional repression in G1 cells but did increase slightly in G2/M cells (Figure 9E). Net1-mCherry signal heterogeneity decreased upon transcriptional repression in G1 and G2/M cells (Figure 9F). In cells containing the pNOY130 plasmid but lacking the single rDNA repeat at chromosome V, repression of transcription from pNOY130 plasmids resulted in Cdc14-GFP signals to form circles (compare to Cdc14-GFP in Figure 7C and Net1-mCherry in Figure 9A) and increased the volume of the Cdc14-GFP signals (compare to Cdc14-GFP in Figure 8A and Figure 9C) (Supplementary Figure S4). Therefore, the single rDNA repeat in chromosome V does not appear to effect expansion of Cdc14 upon loss of transcription. Thus, transcription confines rDNA repeats into a smaller volume and drives the co-localization of rDNA-binding proteins and ribonucleoproteins even though the repeats are not contiguous. These results suggest that, in the absence of rDNA transcription, ribonucleolar proteins localize to a single, homogenous droplet that nucleates rDNA-binding proteins on the surface.

### Removal of R-loops by RNase H1 overexpression alters rDNA locus positioning within nucleolus

**Figure 10.**
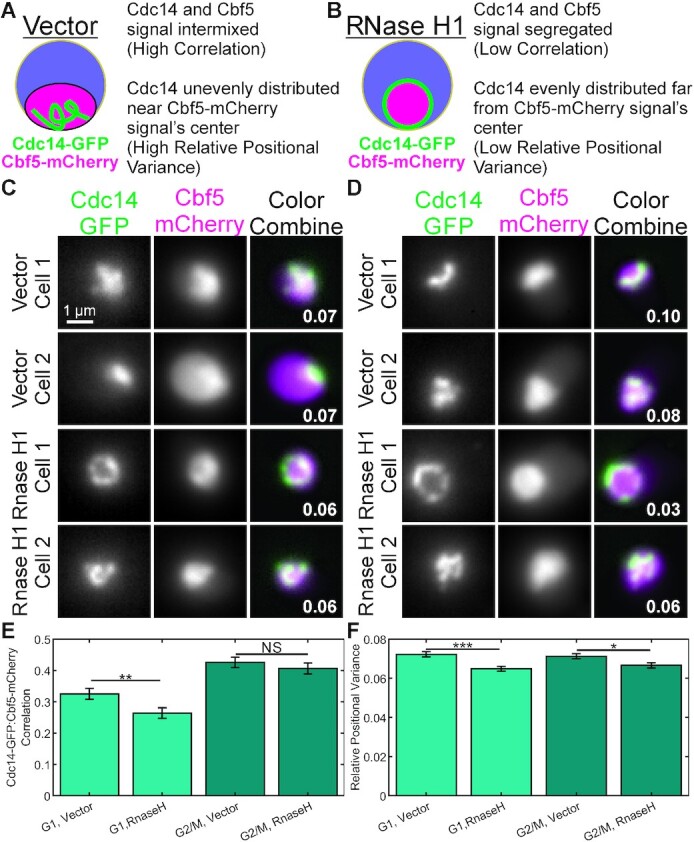
Disruption of R-loops via RNase H1 overexpression displaces Cdc14-GFP from Cbf5-mCherry signals. Schematics of budding yeast nucleoli organization in strains containing a plasmid vector alone (**A**) and a plasmid overexpressing RNase H1 (**B**). Relative positional variance is the variance in the distance of each voxel of the Cdc14-GFP signal from the center of the Cbf5-mCherry signal divided by the mean distance of each voxel of the Cdc14-GFP signal from the center of the Cbf5-mCherry signal ($\frac{{{\sigma ^2}}}{\mu }$). Representative sum intensity projections of budding yeast cells containing the plasmid vector (Vector) or the plasmid overexpressing RNase H1 (RNase H1) in G1 (**C**) or G2/M (**D**). Two cells of each type are shown. White numbers in color combine projections indicate the relative positional variance of the cell shown. Bar charts comparing the Pearson correlation values (**E**) and the relative positional variance (**F**) of Cdc14-GFP and Cbf5-mCherry signals. G1 Vector *n* = 178, G1 RNase H1 *n* = 189, G2/M Vector *n* = 103, G2/M RNase H1 *n* = 94. The *** indicates a *P*-value < 0.001, ** indicates a *P*-value < 0.01, * indicates a *P*-value < 0.05, and NS indicates a *P*-value > 0.05 for Wilcoxon ranksum test.

We wished to determine if the physical co-localization of nascent ribosomal RNA transcripts with the rDNA locus contributed to the transcription-dependent co-localization of the rDNA locus with ribonucleoproteins. R-loops are DNA-RNA hybrid structures and have been shown to form in the rDNA locus (32). RNase H1 specifically degrades R-loops (33) and has been shown to remove R-loops from the rDNA locus of budding yeast *in vitro* (34,35) and in vivo (36–38). We transformed a yeast strain containing Cdc14-GFP and Cbf5-mCherry with a vector control plasmid (pBL189) or with a plasmid expressing the RNase H1 gene Rnh1 (pBB39) on a high copy number plasmid (34) (Figure 10A and B). We observed the same striking separation of Cdc14 signals from Cbf5-mCherry signals upon RNase H1 overexpression than we did for transcriptional repression of pNOY130 (compare Figure 10C and D with Figure 9A and Figure 7B and C). Cdc14-GFP/Cbf5-mCherry signal correlation was significantly decreased upon RNase H1 overexpression in G1 cells but not G2/M cells. However, the positioning of the Cdc14-GFP signals within the Cbf5-mCherry signal was altered in both G1 and G2/M cells (Figure 10F). We then measured the size and position of the Cdc14-GFP signal relative to the Cbf5-mCherry by calculating the relative positional variance (see methods). Cdc14-GFP signals that are intermixed within the Cbf5-mCherry signal have high relative positional variance (Figure 10A), while Cdc14-GFP signals that evenly surround the Cbf5-mCherry signal have low relative positional variance (Figure 10B). We found cells overexpressing RNase H1 had significantly less relative positional variance of their Cdc14 signals than cells containing only the vector (Figure 10F). Thus, upon degradation of R-loops by RNase H1 overexpression, the rDNA locus appears to physically separate from the interior of the nucleolus.

## DISCUSSION

### PPPS vs LLPS condensate

The material properties of the nucleolus reveal that cells can compartmentalize the nucleoplasm into functional subdomains. When nutrients are plentiful the rDNA locus is sequestered within the nucleolus; however, upon nutrient starvation or rapamycin treatment TORC1 is inactivated and the rDNA locus condenses and physically separates from nucleolar proteins during nucleophagy (16) in a process that is dependent on the known DNA looping agents condensin (39) and Hmo1 (17,40). Previous *in silico* studies have shown how the highly looped nature of the rDNA locus could be sufficient to sequester the rDNA locus from the remainder of the genome (6,7). Recently, Erdel and Rippe (11) put forth a set of criteria to describe a biomolecular condensate formed by PPPS. Our study finds the rDNA locus, fluorescently labeled directly with LacI-GFP or indirectly with Cdc14-GFP/mCherry, meets several of these criteria of a PPPS condensate. First, a PPPS condensate would co-localize with chromatin bridging proteins (i.e. condensin and Hmo1). Second, a PPPS condensate can exhibit concentration changes while an LLPS condensate does not. We show the rDNA-binding protein Cdc14 exhibits regional changes in concentration as the fluorescent signal is significantly less homogenous than those of ribonucleoproteins (Figure 2). The rDNA locus itself was shown to be compressible in the population as there was a significant correlation between Cdc14 signals volume and mean intensity (Figure 3). Introduction of additional crosslinks compressed the rDNA locus, via either LacI tetramerization (Figure 4) or recruitment of additional condensin to the rDNA locus (41) upon rapamycin treatment (Figure 5). Last, a PPPS condensate will dissolve if the constitutive chromatin composing the condensate is dissolved. We demonstrate that in a yeast strain containing high-copy rDNA plasmids instead of a contiguous rDNA locus, rDNA-binding proteins Cdc14 and Net1 exhibit a large increase in volume and are excluded from ribonucleoprotein signals (Figures 7-9). Thus, the rDNA locus behaves in a manner consistent with a PPPS condensate.

Surprisingly, the ribonucleoproteins examined in this study did not behave in a manner consistent with a PPPS condensate. Rather, they met the criteria for LLPS condensates (11). The ribonucleoprotein signals appeared homogenous (Figure 2), failed to compress (Figures 3-5) and appeared a singled droplet while excluding rDNA-binding proteins when rDNA transcription was repressed (Figures 7-9). While the budding yeast nucleolus appears to behave as a LLPS condensate in these, the underlying proteins and overall mechanism that drive LLPS of the budding yeast nucleolus to form a biomolecular condensate remains poorly understood.

The exclusion of rDNA-binding proteins from the nucleolus, first observed in TORC1-inactivated cells (16), and again shown in our work upon transcriptional repression suggest the necessity of RNA production for proper encapsulation of rDNA locus by the nucleolus. The decrease in Cdc14/Cbf5 signal correlation and the apparent exclusion of the rDNA locus from the nucleolus upon degradation of R-loops via RNase H1 overexpression (Figure 10) suggests that a physical connection between the rDNA locus and nascent ribosomal RNA transcripts may be necessary to keep the rDNA locus firmly within the nucleolus. These results suggest that encapsulating the rDNA locus within the nucleolus may involve crosslinking between RNA/DNA hybrids formed during transcription and that in the absence of sufficient RNA production, the rDNA can segregate away from the ribonucleoplasm. Our results suggest that the lack of transcription due to condensation of the rDNA locus, not the condensation itself, may push the rDNA from the nucleolus. Recent work using HeLa cell lines has proposed that the exclusion of fully formed ribosomes is due to the loss of interactions of NPM1, SURF6 and other scaffolding components of the granular component matrix with nascent rRNA transcripts as ribosomes mature (14). If nascent rRNA transcripts are hybridized with the rDNA locus and the nascent rRNAs do have multiple interactions sites with ribonucleoproteins then rRNA/rDNA hybrid structures would act to sequester the rDNA locus within the nucleolus in budding yeast.

These studies highlight a potentially novel function for RNA in driving the polymer and liquid phases of the nucleolus together. The concept comes from first principles of macromolecular crowding. A particle is surrounded by what is known as an excluded zone or excluded volume. In the case of crowding, two particles experience an attractive force (depletion force) due to the increase in system entropy that arises when the excluded zones overlap, resulting in a net increase in volume in the system. RNA is excluded from the depletion zone and acts as the osmotic agent in this scenario. It is well known that osmotic agents accelerate reactions such as nucleic acid hybridization (42) and protein crystallization for structural studies. *In vitro* studies with dual optical traps provide direct evidence for the depletion force between two colloids in solutions of nucleic acid (43). The range and amplitude of the depletion force depends on the physical extent of the depletion zone and the pressure exerted by the osmotic agent. It is likely that these depletion interactions contribute to the behavior of complex mixtures of particles and different phase states in the nucleus and provide a new perspective on the role that transcription plays in nucleolar integrity.

## DATA AVAILABILITY

MATLAB analysis programs are available at https://github.com/jlaw8504/nucleolar_quant.

## Supplementary Material

gkab229_Supplemental_File
