# Supplementary material for: The rDNA is biomolecular condensate formed by polymer–polymer phase separation and is sequestered in the nucleolus by transcription and R-loops
Source: Nucleic Acids Res. 2021 Apr 9;49(8):4586–98. doi: 10.1093/nar/gkab229 (PMC8096216; doi:10.1093/nar/gkab229)
Supplement: gkab229_Supplemental_File [file gkab229_supplemental_file.pdf]

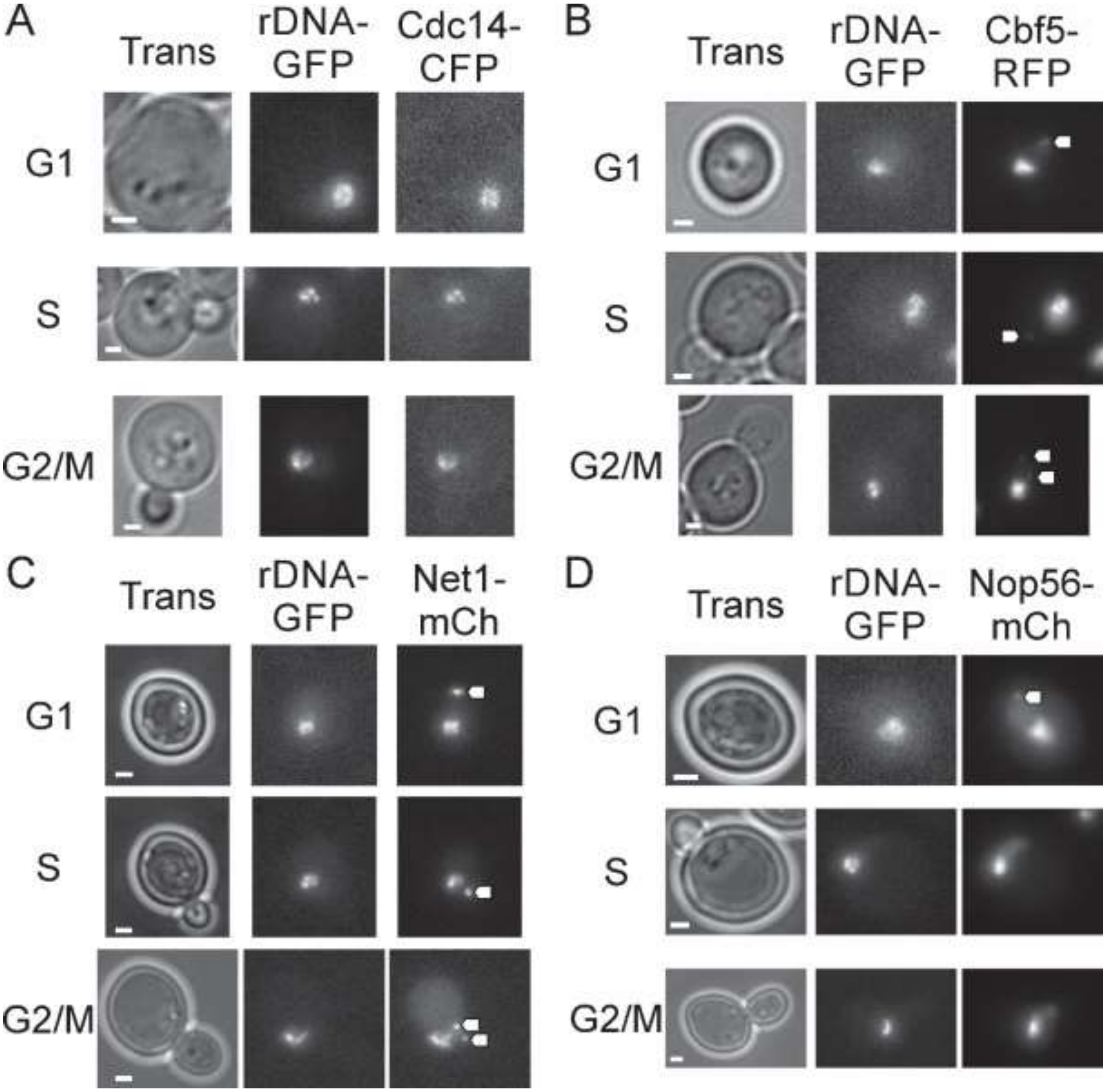

Figure S1. Representative sum intensity projects of entire cells for signals shown in Figure 1. Scale bars are 1 micron. White arrows indicate spindle pole body signals from Spc29-RFP labeling.

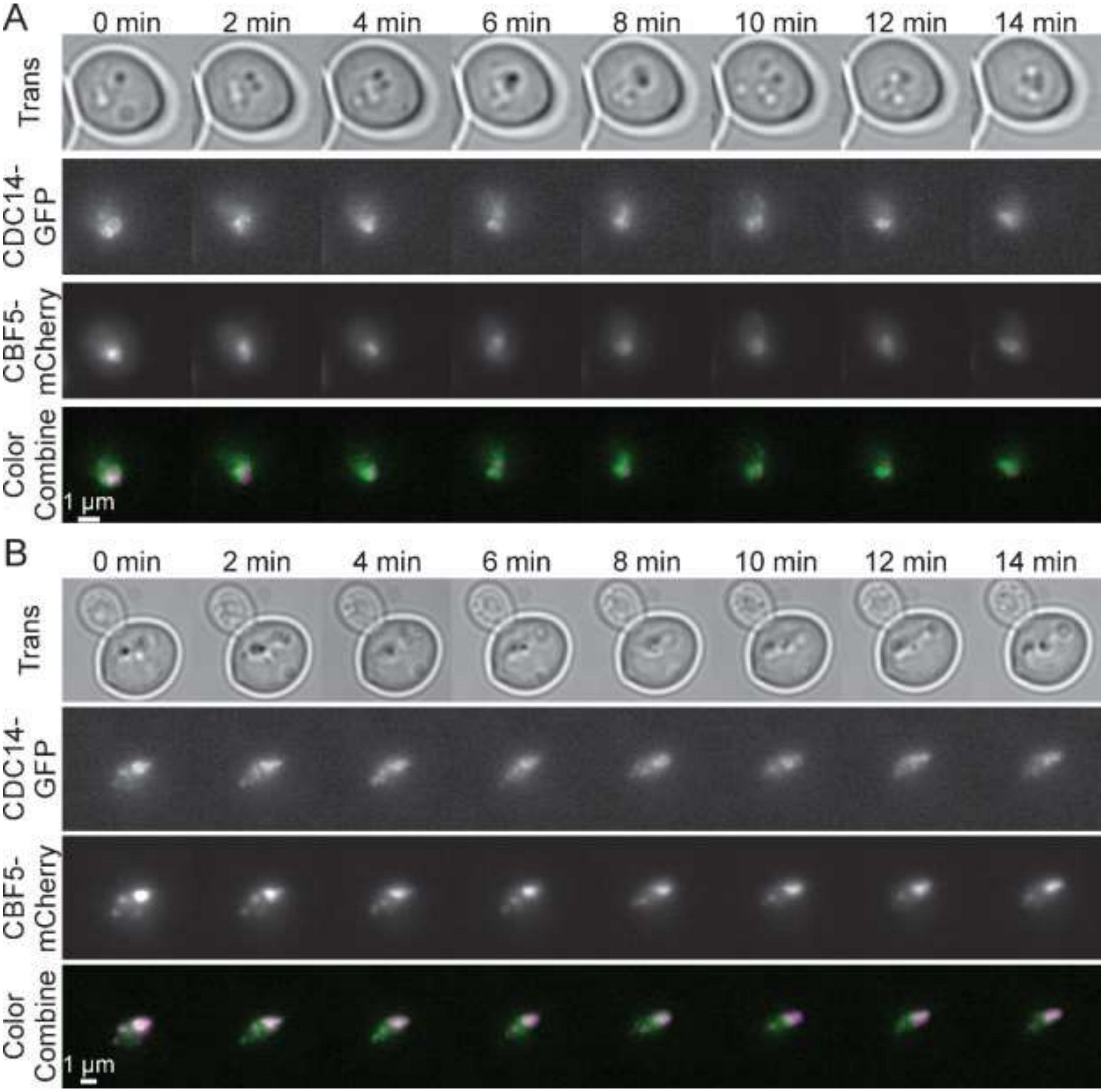

Supplemental Figure 2. Cdc14-GFP and Cbf5-mcherry signal distributions when transcription from pNOY130 is on. Montages of maximum intensity projections of a G1 (A) and metaphase (B) cell expressing Cdc14-GFP (green) and Cbf5-mCherry (magenta). Trans images are single plane. Fluorescent images are maximum intensity projections.

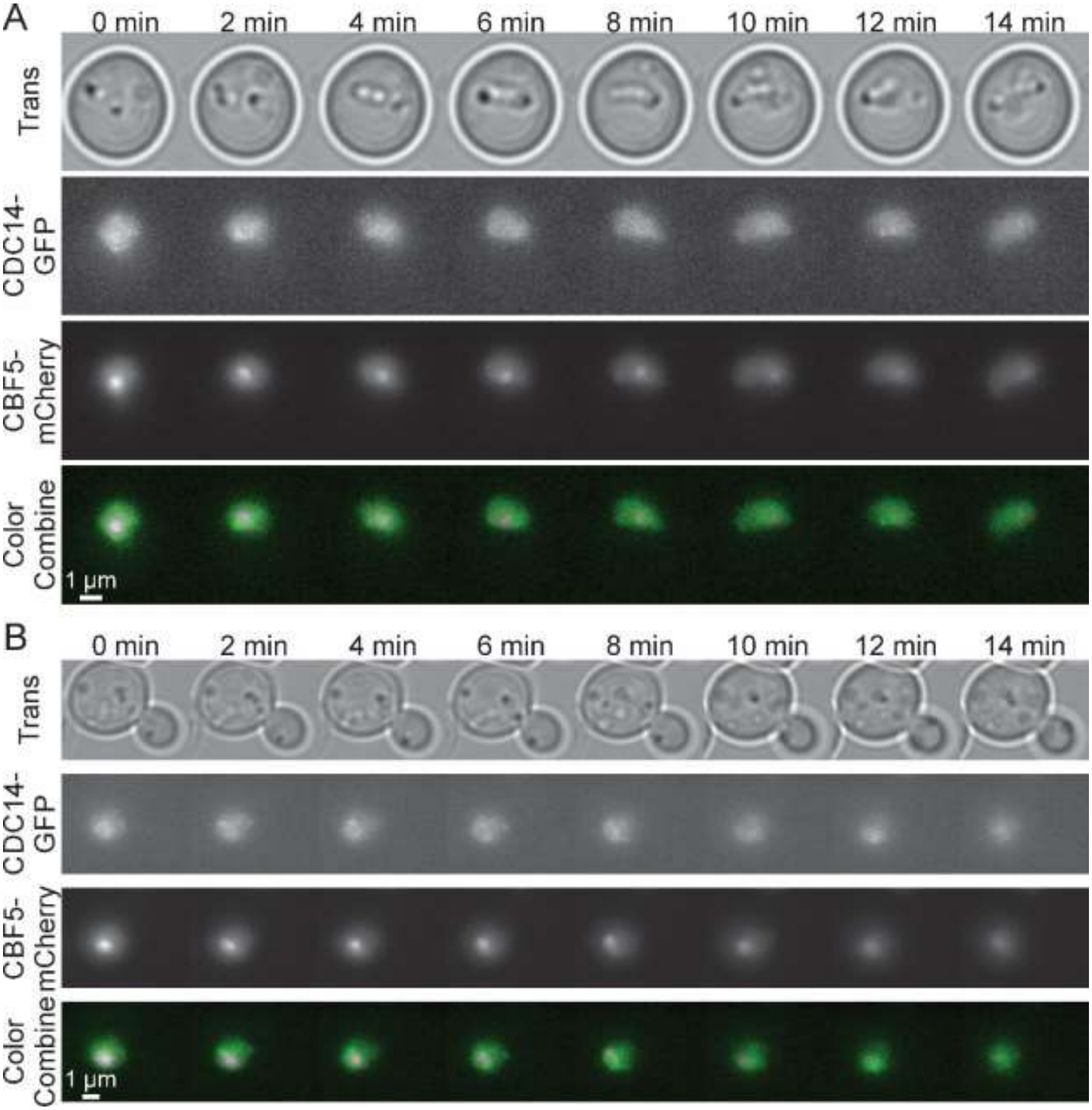

Supplemental Figure 3. Cdc14-GFP and Cbf5-mcherry signal distributions when transcription from pNOY130 is off. Montages of a G1 (A) and metaphase (B) cell expressing Cdc14-GFP and Cbf5-mCherry. Trans images are single plane. Fluorescent images are maximum intensity projections.

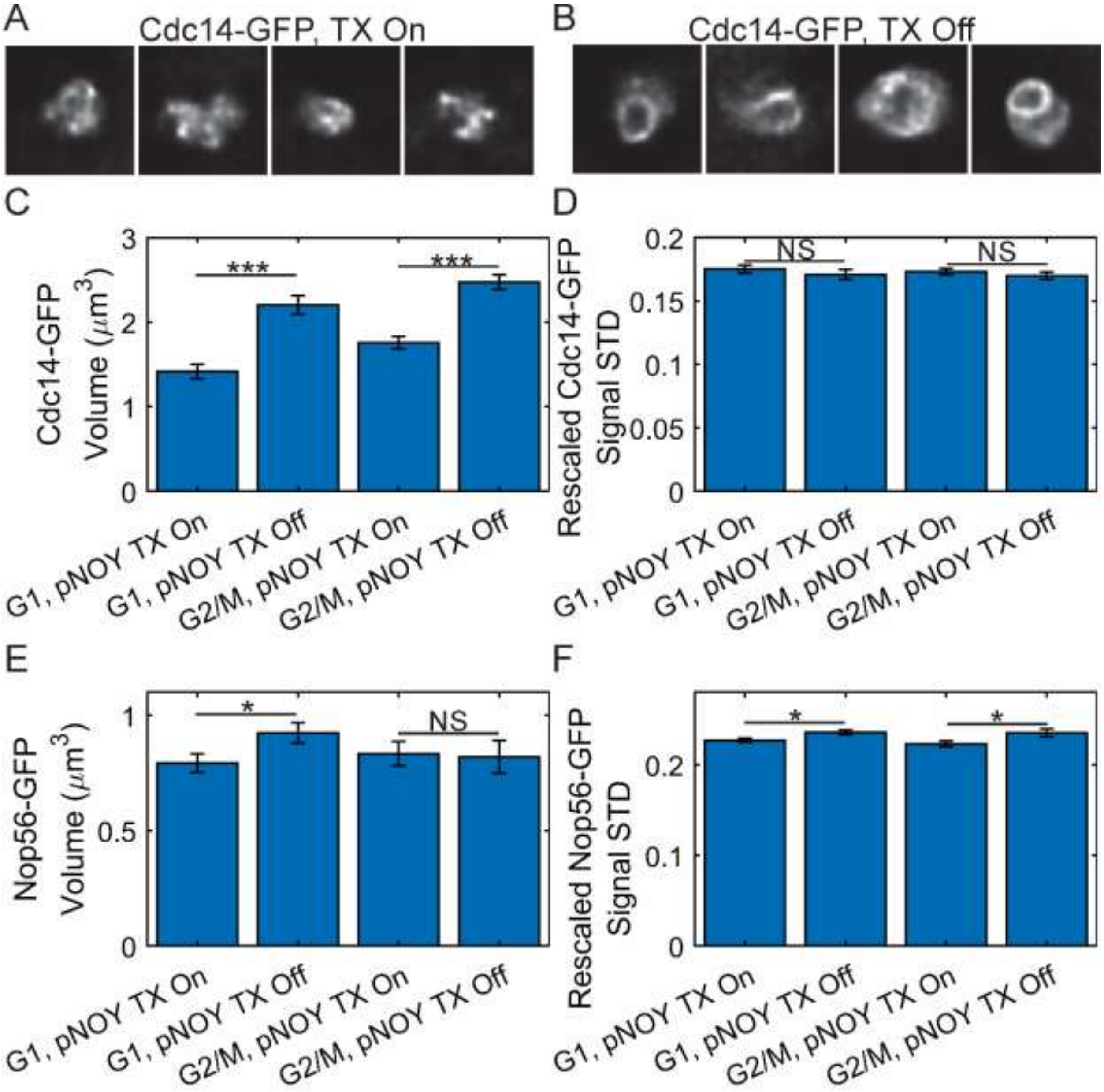

Supplemental Figure 4. Cdc14 signals expand upon loss of transcription in cells containing only pNOY190-based rDNA repeats. Representative images of Cdc14-GFP when pNOY130 expression is on (A) and off (B). Fluorescent images are sum intensity projections of deconvolved z-stacks. Mean volume (C) and rescaled standard deviation (D) of Cdc14-GFP. G1 Tx On n = 46 cells; G1 Tx Off n = 28 cells; G2/M Tx On n = 76 cells; G2/M Tx Off n = 51 cells. Mean volume (E) and rescaled standard deviation (F) of Nop56-GFP. G1 Tx On n = 94 cells; G1 Tx Off n = 79 cells; G2/M Tx On n = 48 cells; G2/M Tx Off n = 26 cells. The *** indicates a p-value < 0.001, * indicates a p-value < 0.05, and NS indicates a p-value > 0.05 for Wilcoxon ranksum test.
